# Microbial Polysaccharides as Functional Components of Packaging and Drug Delivery Applications

**DOI:** 10.3390/polym16202854

**Published:** 2024-10-10

**Authors:** Aigerim Yermagambetova, Sagdat Tazhibayeva, Paul Takhistov, Bakyt Tyussyupova, José Agustín Tapia-Hernández, Kuanyshbek Musabekov

**Affiliations:** 1 Faculty of Chemistry and Chemical Technology, Al-Farabi Kazakh National University, Almaty 050040, Kazakhstan; tazhibayeva_s@mail.ru; 2 Department of Food Science, Rutgers State University of New Jersey, New Brunswick, NJ 07102, USA; ptakhist@sebs.rutgers.edu; 3 Department of Chemical Technology and Chemistry, Kazakh-British Technical University, Almaty 050000, Kazakhstan; musabekov40@mail.ru; 4 Departamento de Investigación y Posgrado en Alimentos (DIPA), University of Sonora, Hermosillo 83000, Sonora, Mexico; joseagustin.tapia@unison.mx

**Keywords:** microbial polysaccharides, biodegradable packaging, antimicrobial activity, drug delivery

## Abstract

This review examines microbial polysaccharides’ properties relevant to their use in packaging and pharmaceutical applications. Microbial polysaccharides are produced by enzymes found in the cell walls of microbes. Xanthan gum, curdlan gum, pullulan, and bacterial cellulose are high-molecular-weight substances consisting of sugar residues linked by glycoside bonds. These polysaccharides have linear or highly branched molecular structures. Packaging based on microbial polysaccharides is readily biodegradable and can be considered as a renewable energy source with the potential to reduce environmental impact. In addition, microbial polysaccharides have antioxidant and prebiotic properties. The physico-chemical properties of microbial polysaccharide-based films, including tensile strength and elongation at break, are also evaluated. These materials’ potential as multifunctional packaging solutions in the food industry is demonstrated. In addition, their possible use in medicine as a drug delivery system is also considered.

## 1. Introduction

The problems of managing plastic waste and its uncontrolled accumulation in the ecosystem cause serious environmental difficulties [1,2]. The persistence of plastics in the environment leads to pollution and harming wildlife and ecosystems, and contributes to the global plastic pollution crisis. Addressing these issues requires innovative solutions in materials science, including developing biodegradable materials for various applications, including food packaging and drug delivery.

For this reason, there are several methods by which food packaging materials can be produced using microbial polysaccharides. Such packaging materials decompose rapidly and are readily accessible [1,3,4].

Due to their diverse properties, including viscosity, rheological characteristics, and the ability to swell and interact with specific structures, microbial polysaccharides have a wide range of applications [5]. For example, gellan gum is used to produce plasmid DNA, which is currently being investigated for treating human papillomavirus [6], and pullulan is used as a nanomaterial [7]. Microbial polysaccharides have the potential to be employed as a material for the fabrication of bandages for use on blood vessels and internal organs [8]. In the food industry, microbial polysaccharides are used for the production of textured fruits [9] and for the production of frozen products, e.g., frozen cooked noodles [10], as well as for the production of fermented dairy products such as yogurts [11]. In the cosmetics industry, microbial polysaccharides are used to increase dispersions’ stability, improve creams’ texture, and increase biodegradability [12]. Viscous solutions and dry films based on conventional polysaccharides are practically impossible to use in technical or medical materials due to their poor quality and mechanical properties.

As shown above, microbial polysaccharides have a wide range of applications in various industries. Especially in medicine, their potential for use is considerable, making them suitable for drug delivery. The advantages of microbial polysaccharides, including biocompatibility [13,14], renewability [15], and the ability to form stable gels [16], make them an ideal material for the prolonged delivery of medicinal forms. In medical applications, these biopolymers can be engineered to control the release of drugs, enhance the stability of medicinal compounds, and improve patient outcomes by providing targeted and sustained delivery.

Microbial polysaccharides are extensively employed for food packaging due to their functional properties [16] and ability to synthesize them on an industrial scale. 

One of the disadvantages of microbial polysaccharides is that they lack sufficient durability compared to non-degradable packaging materials. Nevertheless, in light of the environmental issues associated with the accumulation of non-degradable polymer packaging, microbial polysaccharides should be regarded as a viable alternative.

Despite the clear advantages of using microbial polysaccharides, there is little literature on the complex analysis of their physico-chemical properties. A comparative analysis of their properties would facilitate a more targeted regulation of their properties, depending on the field of application. This study aims to summarize the research results on using microbial polysaccharides in different industrial sectors, including medicine, and describe their advantages and disadvantages compared to commonly used polymers. The objective is to demonstrate the prebiotic and antioxidant properties of microbial polysaccharides as well as their role in the drug delivery system. Furthermore, this study aims to identify future applications of microbial polysaccharides and their potential to increase efficiency and environmental sustainability. 

## 2. Physico-Chemical Properties of Major Microbial Extracellular Polysaccharides

Polysaccharides are a ubiquitous component of natural ecosystems. Bacteria produce them and have special properties. The structure of microbial polysaccharides can be either linear or highly branched and consists of sugar residues linked by glycosidic bonds. There are two types of exopolysaccharides: heteropolysaccharides, such as D-glucose, D-galactose, and L-rhamnose; and homopolysaccharides, such as α-D-glucans, fructans, and β-D-glucans [17]. These are non-toxic compounds obtained by periodic aerobic immersion fermentation in two forms: spherical and encapsulated polysaccharides [18]. The principal factor in selecting microbial polysaccharides as a packaging material is their physico-chemical and film-forming properties. It is noteworthy that the materials in question exhibit viscosity at low polymer concentrations, as well as notable mechanical strength, elasticity, and biocompatibility.

Currently, a considerable number of polysaccharides of microbial origin are known, as shown in Table 1.

Microbial polysaccharides can form elastic films when combined with other polymers. For instance, the elongation at break of a film made of 1% agar–gellan gum by weight is 25.9% ± 0.9 [34] while the same for a film made of 1% chitosan–curdlan gum by weight is 8.81% ± 0.38 [35]. In comparison, a film composed of 4% cassava starch and xanthan gum by weight has an elongation at break of 17.1% ± 3.5 [36]. These data suggest that gellan gum can form more elastic films than curdlan gum and xanthan gum.

### 2.1. Xanthan Gum

Xanthan gum is a widespread microbial heteropolysaccharide produced by *Xanthomonas campestris* during fermentation and is found on the leaves of plants belonging mainly to the cabbage family. *Xanthomonas campestris* is a bacterium that belongs to the group of gamma-proteobacteria. The genus *Xanthomonas* is aerobic, yellow-pigmented, and gram-negative [33,37,38]. 

Xanthan gum has considerable potential for commercial applications, especially in medicine, due to its exceptional matrix-forming properties and high biocompatibility [33]. Xanthan gum has various molecular weights and properties and can form a robust and resistant structure, making it an ideal protein carrier and drug delivery system [39]. The rheological properties of xanthan gum are very attractive for various applications exhibiting high viscosity even at a concentration of ≤1% [40]. It is widely used in foods (such as dairy products, bakery products, and beverages), pharmaceuticals, and cosmetics [41]. 

Studies have confirmed that xanthan gum has excellent stabilizer and water-binder properties that contribute to improved texture and controlled release of flavors in various foods [42]. Xanthan gum is not only used in cosmetics and personal care products to improve rheological properties. It is also used as an ingredient that facilitates the formation of a protective film in moisturizers, thereby improving the moisture properties of the skin [43]. This was demonstrated in [44] by a thermal analysis method using elevated temperature and an increase in the concentration of xanthan gum. At a temperature of 100 °C, with an increase in the concentration of xanthan gum, the polymer’s ability to bind water also increases, which can lead to an increase in porosity.

### 2.2. Pullulan

Pullulan is a water-soluble polysaccharide obtained extracellularly from *Aureobasidium* pullulans [45]. *Aureobasidium* sp. fungi, known as black yeast due to melanin production, are cosmopolitan [46,47]. Compared to other polysaccharides, pullulan films have a significantly lower oxygen permeability [46], are colorless, transparent, odorless, and tasteless. However, the hydrophilicity and stiffness of pullulan films may limit their use in industry [48].

Consequently, pullulan has considerable potential in several industrial biotechnology sectors [49]. Currently, pullulan is used by numerous biotechnology companies for the production of innocuous pharmaceutical capsules in the medical industry and as an essential ingredient in cosmetics [50,51]. In this work [50], pullulan films with encapsulated extracts were prepared, which exhibited excellent rheological properties due to the inherent qualities of pullulan. The fragility of pure pullulan film renders it unsuitable for use as a packaging material in the work [52]. Consequently, the incorporation of lactate nanocellulose enhanced the tensile strength of the film (TS) from 43.7 MPa to 76.6 MPa.

The main advantages of pullulan are that it is a non-ionic microbial polysaccharide which is compatible with blood, biodegradable, non-toxic, non-immunogenic, non-mutagenic, and non-carcinogenic [53]. Therefore, it can be used in producing food films [39] and for producing antimicrobial membranes that promote wound healing [54], where pullulan has shown good biocompatibility with various materials.

Like many other films based on microbial polysaccharides, pullulan-based films have some disadvantages. They have hydrophilic properties, are water-soluble, have a relatively short life span, and can decompose quickly. Polyethylene is more often used as a packaging material than pullulan-based films. Therefore, the development of innovative food packaging films based on a combination of pullulan and a synthetic polymer has a basis and is relevant [55]. 

### 2.3. Curdlan

Curdlan, an extracellular polysaccharide, is fermented in particular by bacteria of the *Rhizobium* family [23], which are considered non-pathogenic and non-toxic microorganisms. Curdlan is soluble in an alkaline solution but not in water or alcohol. It is important to emphasize that a gel may form in the water dispersion system when curdlan is heated. Curdlan is used in the production of a wide range of foods, including tofu, noodles, jelly, and low-fat meat alternatives [56]. As one of the members of the pyridoxal polysaccharide family consisting of D-glucose linked by 1,3-β-glycosidic bonds, it is non-toxic, biodegradable, chemically modeled, and processed for recycling [57,58,59]. The formation of pure curdlan films has been observed to occur with good tensile strength, with a recorded value of 4.53 MPa. However, in the work [52], walnut green husk polyphenol improved hardness, reaching 111.83 MPa.

Curdlan is insoluble in most solvents, including ethanol and water. However, it may dissolve in alkaline aqueous solutions with a pH above 12 due to changes in its conformation. In the food industry, curdlan has been widely used as a food additive in recent years due to its high-temperature resistance, safety, and non-toxicity [23,60].

Apart from the fact that curdlan is used in the food industry in some countries [61], it is an effective reagent in the production of heavy concrete, preventing the layering of cement and small stones [60], and is also used as an organic binder in ceramics [57]. In addition, the antitumor properties of curdlan are currently being investigated in medicine [23]. This phenomenon can be attributed to curdlan having optimal carrier properties for both RNA and chemotherapeutic agents [62]. The nanoparticles in question have several fundamental properties, including the ability to bind to miRNA and protect against enzymatic degradation and the phagocytic system that maintains stability under physiological conditions. They also improve intracellular uptake and support the release of miRNA and drugs [63]. Curdlan can activate dendritic cells and improve the antitumor immunity of cells [64]. However, curdlan’s hydrophobicity prevents its optimal binding to receptors. In this context, research is currently underway to obtain curdlan derivatives, including the production of oxidized curdlan [65], sensitive curdlan-based polymers [62], and curdlan nanoparticles [66]. The production of such derivatives requires urgent research in the fight against cancer cells and could become a discovery that will change medicine [67].

### 2.4. Bacterial Cellulose

Bacterial cellulose is produced by a variety of microorganisms, including tunicates, certain species of algae and various aerobic, non-pathogenic bacteria belonging to the genera *Acetobacter*, *Agrobacterium*, *Pseudomonas*, *Rhizobium*, and *Sarcina*. Bacterial cellulose is synthesized from glucose in the form of a pellicle and extruded through pores in the cell wall. The glucose chains are converted into microfibrils that aggregate into cellulose ribbons [68]. A cell-free enzyme system has also been developed for the synthesis of bacterial cellulose, which has significant potential for industrial-scale production. The cell-free enzyme system is based on bacterial cellulose-producing strains and contains all the necessary enzymes and cofactors for synthesis [69]. This process is based on continuous production and allows for the circumvention of restrictions on cell growth and viability at elevated temperatures [70]. This system was developed based on a single cell line, using a cost-effective and straightforward approach. The results demonstrate a notable yield [71]. Furthermore, the physico-chemical properties of cellulose obtained by synthesis based on a cell-free system are superior to those of cellulose based on microbial polysaccharides [72]. To illustrate this, the strength of cellulose derived from a cell-free system (17.63 MPa) is superior to that of bacterial cellulose (14.71 MPa) [73]. This quality may be advantageous or disadvantageous, contingent on the intended application.

Bacterial cellulose is considered a natural polymeric hydrogel with three-dimensional polymer networks, flexibility, and good mechanical properties. It can change its size, shape, and properties in response to fermentation conditions. The presence of the Van der Waals force facilitates the formation of crystalline nanofibers, which subsequently give rise to a microfibrillar structure. The presence of a superspiral structure contributes to observing hierarchical orders, which provide cellulose with a high degree of mechanical strength [74]. It has several advantages over cellulose regarding plant origin, including high purity, moisture permeability, mechanical strength, non-toxicity, environmental friendliness, biocompatibility, and biodegradability [3,22]. Bacterial cellulose has a number of properties that make it suitable for use in a variety of fields. In addition, the structure of bacterial cellulose depends on many factors and may differ from one another [75].

Due to its numerous advantages, bacterial cellulose has considerable potential in a wide range of industries. These include applications in food packaging [76], coatings or films [4], absorbent materials [77], medicine [78], pharmaceuticals [79], biomaterials [80] and the electronics industry [81]. A novel cellulose-based metal–organic framework, created through the use of 3D printing technology, was recently introduced. This framework comprised oxidized cellulose nanofibers, which were oxidized using a 2,2,6,6-tetramethylethyl piperidine-1-oxyl radical. Incorporating these components with zeolitic imidazolate frameworks represents a significant advancement in the 3D printable hydrogel ink field, with potential in biomedical applications. The release of model drugs (curcumin and methylene blue) from printed scaffolds has demonstrated the ink’s capability to facilitate the controlled release of drugs [79]. Bacterial cellulose can be synthesized using three different methods: the static method, the stirred/shaken culture method, and cultivation in a bioreactor [82]. Bacterial cellulose, for example, is superior to plant cellulose in the production of packaging material. This is due to its higher degree of crystallinity, purity, mechanical strength, and ability to bind moisture [83]. The strength of the bacterial cellulose film is quite high at 119.56 ± 7.56 MPa. It is higher than cellulose film/chitosan nanoparticles (45.39 ± 2.80 MPa). The elongation at the break of the bacterial cellulose film (17.5%) is also higher than that of the cellulose film/chitosan nanoparticles (4.5%) [84].

This renders it unsuitable for use as a food packaging material [15]. Nevertheless, study [85] demonstrated that the incorporation of bacterial cellulose with gellan gum enhanced the vapor permeability of the film 0.429 g·mm·m^−2^·d^−1^·kPa^−1^, in contrast to the film based on pure bacterial cellulose (3.6 times lower).

The nanocrystals produced by bacterial cellulose can also be used as a nano-reinforced material for the production of low-cost, lightweight, and highly durable nanocomposite films that perform better than polymers [86].

A study of the membrane made with bacterial cellulose and phenol-based ionic liquids has shown the potential for anti-inflammatory, antioxidant, and non-cytotoxic properties [87].

## 3. Barrier Properties of Microbial Polysaccharides

Water vapor permeability (WVP) is used to measure water vapor transmission through different materials. It is measured in accordance with the European standard ASTM E96-95 [88]. In food packaging, the hydrophobicity of materials is a crucial factor to consider, as it can affect the product’s properties, regardless of whether the product is sensitive to water. It is recommended that food packaging has a low WVP value, which reduces and prevents the transfer of moisture between the product and the environment. In this context, the vapor permeability of the film depends on the composition and type of polymer material [89]. The WVP’s dependence on the films’ content and their concentration is shown in Table 2.

The data presented in Table 2 show that films containing bacterial cellulose and two or more microbial polysaccharides are the most susceptible to water vapor, or, in other words, the most breathable. This is a significant disadvantage for food storage [68]. The polysaccharides pullulan and xanthan gum allow less air to pass through than other polysaccharides, which is their advantage. Adding the microbial polysaccharide xanthan gum to the composition of food packaging also reduces its WVP [95]. In addition, among the polysaccharides and their compositions, a mixture of xanthan gum and alginate with pectin, pullulan, and carrageenan has a fairly high water vapor resistance, surpassed only by pure pullulan [96]. It is obvious that by changing the ratio between the amount of microbial polysaccharides and biopolymers, it is possible to obtain compositions with the required properties of vapor permeability and elastic strength.

## 4. Biodegradability of Microbial Polysaccharide Materials

The main advantage of microbiological polysaccharide materials is that they are biodegradable and do not contribute to environmental pollution [97].

The biodegradation of packaging materials is usually assessed by quantifying the conversion of organic carbon to CO_2_ in closed vessels under controlled laboratory conditions [98]. The European standard EN 17033 specifies the requirements for biodegradable films made from thermoplastic materials to be used for mulching applications in agriculture and horticulture, which deals with the use of biodegradable plastic films in agriculture and horticulture, stipulates that at least 90% of the organic carbon present in the plastic must be converted to CO_2_ within two years when incubated in soil or in combination with a biodegradable reference substance such as cellulose or poly-(3-hydroxybutyrate) [99]. The possibility of using the FT-IR spectroscopy method to monitor the biodegradation of starch and agar-based films is also shown [100]. For this purpose, it is necessary to record the changes in the FT-IR spectra of the films over a certain period of time.

At present, biodegradable films from renewable sources cannot yet fully replace the synthetic plastic food packaging currently available on the market. In certain cases, these materials allow the regulation of gas exchange, moisture, and the migration of undesirable aromas and/or compounds. However, they also introduce an additional stress factor for food preservation [1].

The use of packaging based on microbial polysaccharides has the advantage of being biodegradable and serving as a renewable energy source, thereby reducing environmental impact [101]. The rate of biodegradation depends on the molecular weight of the polymer and/or its composition (e.g., the presence of plasticizers, dyes, antipyretic agents), the presence of microorganisms, and environmental factors such as humidity, temperature, and the presence of microorganisms [97]. The large macromolecules of most polymers are usually unable to penetrate cell membranes. Microorganisms use plastic as a carbon source in the absence of other nutrients [102]. Environmental factors such as temperature, humidity, and decomposing microorganisms in the atmosphere and soil greatly influence the decomposition of biodegradable packaging materials. Climatic conditions, weather variables, and soil composition influence these factors. Consequently, biodegradable packaging materials might decompose completely in one environment while showing only slight decomposition in another setting [103]. 

Films made from microbial polysaccharides have not been widely used in industry. Moreover, microbial polysaccharide-based films have lower barrier and mechanical properties compared to polymer films. This is because they are less resistant to high humidity inside the product and the surrounding environment.

**Figure 1:**
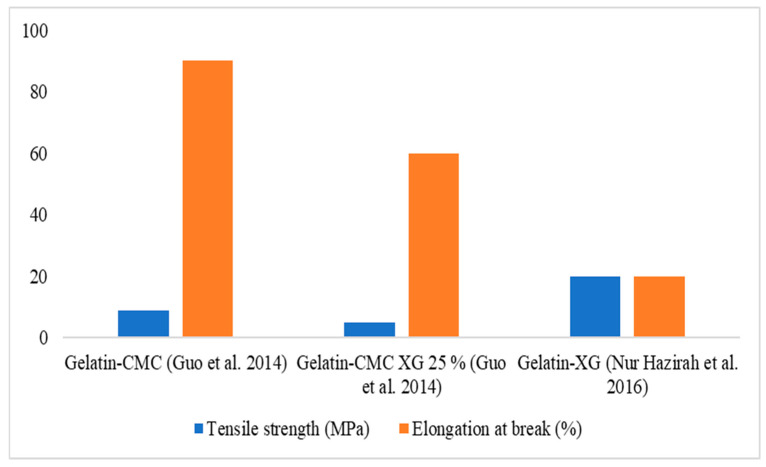
Tensile strength and elongation of break of xanthan gum films with added gelatin [104] and carboxymethyl cellulose [105].

It is important to note that xanthan gum cannot form a film on its own [95]. However, it can enhance the rheological properties of films based on polysaccharides when combined with them. For example, xanthan gum can improve the tensile strength and decrease the elongation of films at the breaking point, as shown in Figure 1.

Using xanthan gum in industrial applications has some physico-chemical limitations, prompting current attention to modified xanthan gum production to address these drawbacks [105,106].

Pullulan films are limited by undesirable properties such as poor mechanical characteristics, high hydrophilicity, and a lack of active functions [107]. Recent studies have shown that the combination of pullulan and starch can significantly improve the physical properties of the starch film. For example, incorporating pullulan into the starch film increased its tensile strength [48]. Increasing the proportion of pullulan in the composite film reduced tensile strength but simultaneously enhanced elongation at break. Additionally, the optimal ratio of starch and pullulan improves the moisture permeability of the packaging material [108]. Pullulan-based films are edible, uniform, transparent, heat-sealable, elastic, provide good oxygen barriers, have no taste or odor, are non-toxic, and are biodegradable [109]. The primary commercial use of pullulan is in producing edible films used in various breath fresheners or oral hygiene products [46].

**Figure 2:**
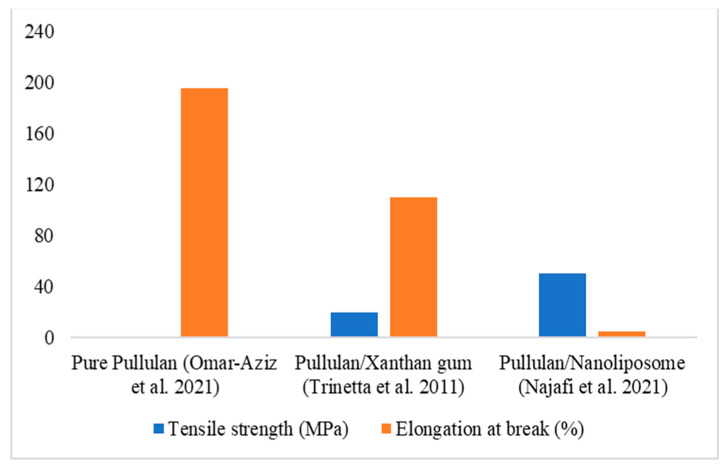
Tensile strength and elongation of break pullulan with xanthan gum and nanoliposome [50,110,112].

Pullulan has excellent film-forming ability. However, pure pullulan film is highly elastic but has low strength [110]. Therefore, it is essential to incorporate certain additives to enhance the tensile strength of pullulan films. For instance, xanthan gum has been shown to improve the tensile strength of pullulan films [111]. As shown in Figure 2, nanoparticles/nanoliposomes are used to modify the rheological properties of the pullulan film [50].

Curdlan has garnered significant interest in the food industry due to its unique physical and chemical properties. As a neutral microbial polysaccharide, curdlan does not dissolve in water or alcohol [113]. Therefore, when dispersed in aqueous solutions, it can facilitate the formation of gels with improved quality and mechanical properties. Additionally, curdlan can induce gelation when the solution’s alkalinity is neutralized by acids in a static medium [114]. Currently, the successful production of mixed films of curdlan with nanocellulose [68], curdlan with polyvinyl alcohol [61], and curdlan with chitosan [35] have demonstrated promising results, including enhanced thermal stability and water-resistant properties. Consequently, curdlan can be considered a valuable composite material for enhancing other hydrophilic biopolymers’ mechanical, thermal, and water-resistant properties [62].

## 5. Microbial Polysaccharides as Functional Materials: Antimicrobial, Antioxidant, and Prebiotic Properties

The packaging of food products is designed to extend their shelf life and preserve their quality. In addition to these properties, biodegradable controlled-release packaging is also created with antioxidant and antimicrobial functions in mind [115]. Microorganism growth can cause food spoilage and compromise its safety. The oxidation of lipids, proteins, vitamins, and pigments contributes to losing nutrients and quality, including denaturation and discoloration [91].

Microbial polysaccharides can also act as antimicrobial agents. The length of a microbial polysaccharide chain and its molecular weight influence its biological activity, including its antimicrobial activity. Antimicrobial activity is increased due to viscosity-related biological activity. The mechanisms by which polysaccharides affect microbial pathogens and the immune system are complex and not yet fully understood [18].

Microbial polysaccharides have shown antimicrobial action against pathogens such as Shigella sonnei, Cronobacter sakazakii, Staphylococcus aureus, Escherichia coli, Salmonella typhimurium, Bacillus cereus, Listeria monocytogenes, and Candida albicans. Most researchers have suggested that antimicrobial packaging is more effective against gram-positive than gram-negative bacteria [116]. Additionally, modifications of microbial polysaccharides or their mixtures with several additives are more effective than pure microbial polysaccharides, which is illustrated in Table 3.

The use of microbial polysaccharides may not require direct contact, while a non-volatile substance may come into direct contact with the surface of food products. The release and diffusion of active compounds through polymer layers are important factors in the antimicrobial activity of the substance. An antimicrobial substance tightly bound in packaging matrices has a limited release and, therefore, does not inhibit microorganisms [124]. 

The antimicrobial activity of biodegradable films is usually tested using standard methods for enumerating bacteria in food products. The results of the antimicrobial activity study, which are obtained by counting the number of colonies of inactive packaged foods, may differ from the results of the in vitro study. For example, the temperature difference between food storage conditions and the in vitro incubation of microorganisms may affect the antimicrobial efficacy of these films [125].

The antimicrobial compounds incorporated into packaging materials for food products are designed to maintain their concentrations for the duration of long-term storage [126].

Antioxidants are substances that can slow down the oxidation process and neutralize oxidative radicals. Free radicals in the body can damage important biomolecules such as lipids, proteins, and DNA, leading to the development of chronic diseases such as atherosclerosis, cancer, diabetes, cardiovascular diseases, chronic inflammation, stroke, and septic shock. Oxidative stress can also speed up the aging process and contribute to the development of degenerative diseases. The antioxidant activity (DPPH radicals) of microbial polysaccharide modifiers or additives is greater than that of pure microbial polysaccharides, as shown in Table 4 [11].

Microbial polysaccharides have been suggested to have antioxidant properties and could be used in treating tumors. They offer several biotechnological advantages, including a quick fermentation process and easy production of stable emulsions. Additionally, these polysaccharides have minimal side effects and cytotoxicity, making them promising candidates for use in immunotherapy as antioxidants and antitumor agents. By eliminating free radicals, microbial polysaccharides can effectively act as powerful antioxidants [130].

**Figure 3:**
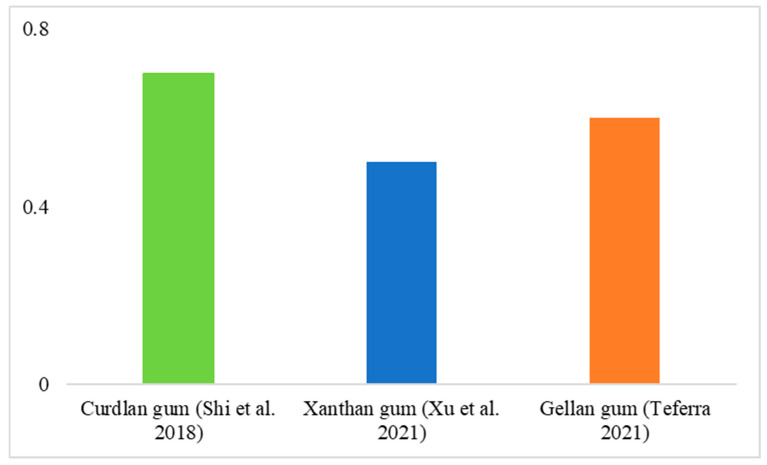
Prebiotic activity score (PAS) of microbial polysaccharides *Lactobacillus casei* [132,133,134].

**Figure 4:**
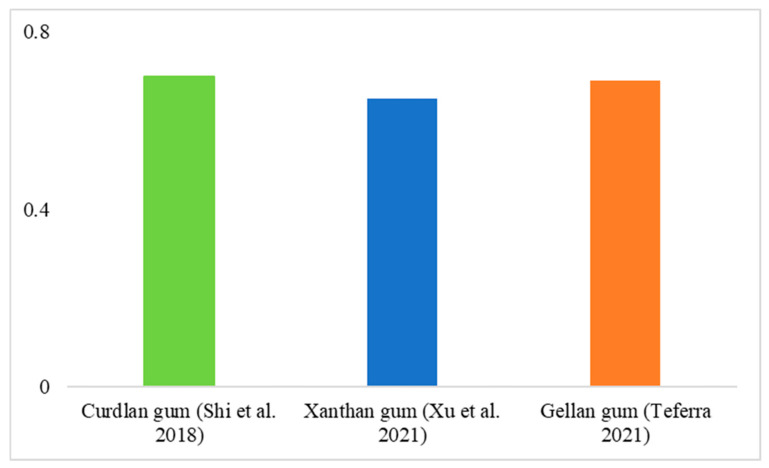
Prebiotic activity score (PAS) of microbial polysaccharides *Lactobacillus rhamnosus* [132,133,134].

It is currently estimated that a significant proportion of the global population is affected by severe various health issues related to the gastrointestinal tract. Prebiotics are carbohydrates that cannot be digested by the human body and are instead fermented by beneficial bacteria in the colon. According to the definition, probiotics should first be tolerant to low gastric pH and resistant to digestive enzymes in the small intestine. Once in the colon, prebiotics can be fermented by the intestinal microbiota of the gastrointestinal tract, thereby stimulating the growth of certain local intestinal bacteria, such as bifidobacteria and lactobacilli [131]. The impact of multiple microbial polysaccharides on the activity index of *Lactobacillus* is illustrated in Figure 3 and Figure 4, where xanthan gum produced by *Xanthomonas campestris,* curdlan gum produced by *Rhizobium,* and gellan gum produced by *Sphingomonas elodea* are compared.

Prebiotics are substances that are fermented by probiotic bacteria in the lower intestine. They become available to other beneficial bacteria in the gut without being absorbed by other intestinal bacteria. Some examples of prebiotics commonly used in the human diet are lactulose, galactooligosaccharides, fructooligosaccharides, cyclodextrins, insulin and its hydrolysates, maltooligosaccharides, and resistant starch [134]. The probiotic activity index is a measure that quantifies the extent to which prebiotics can promote the growth of intestinal bacteria. Studies have found that curdlan, a newly identified prebiotic, has prebiotic effects that enhance the growth of *Bifidobacterium* sp. in the cecum of a rat. When rats were fed curdlan from the bacterium *Rhizobium* for four weeks, their feces significantly increased the bifidobacteria population [132]. Therefore, it is essential to consider the antimicrobial, antioxidant, and prebiotic properties of antimicrobial polysaccharides. Biodegradable films can be used for packaging food or as a thin coating on or between food items. Edible coatings are a layer of food material on the food product itself. The thickness of food films is less than 0.3 mm, and they wrap around food products. Food coatings are created by immersing products in liquid solutions of food materials [135]. Microbial contamination is a significant factor contributing to the deterioration of food. Traditional food preservation methods include fermentation, drying, the addition of antimicrobial agents (such as organic acids, plants, and salts), heat treatment, freezing, cooling, and irradiation. However, these methods have inherent limitations and disadvantages, especially when used with fresh meat [66]. To address these challenges, a range of packaging systems with different properties and applications have been developed as an alternative to traditional food preservation methods. Conventional packaging is meant for short-term storage in a refrigerator, while modified atmospheric packaging or vacuum packaging is used to extend the shelf life of products [91].

## 6. Antimicrobial Packaging

Antimicrobial packaging (AM) is a type of active packaging. In this type of packaging, an antimicrobial compound is added to the packaging material to reduce or prevent contamination of packaged foods by microorganisms that spoil food [136]. The active packaging family includes AM agents used to create the AM packaging. Using AM packaging has several advantages over directly adding AM agents to food products. This is because applying AM agents to the surface of products through spraying or droplets is not effective enough in suppressing microorganisms. This is due to the rapid diffusion of the AM agent into the food and the denaturation of active substances by food components, reducing AM agents’ reactivity [137].

Their advantage over other polymers or synthetic materials lies in their potential for industrial-scale production. However, achieving this is not a simple process. It requires the use of sophisticated machinery, specific substrates, a reliable supply of energy and water, and highly skilled personnel [56].

Microbial polysaccharides produced by *Bifidobacterium longum* have been shown to impair the cell division of microbial pathogens, such as *Vibrio parahaemolyticus* and *S. typhimurium*. Additionally, microbial polysaccharides produced by *Lactobacillus gasseri* exhibit antimicrobial activity against several pathogens, including *Listeria monocytogenes* [18]. Similarly, the pullulan derivative backbone demonstrates antimicrobial activity against *Escherichia coli* and *Staphylococcus aureus* [55]. Furthermore, cryogel, developed using bacterial cellulose, has demonstrated an antimicrobial effect and can control the release of the pesticide tebuconazole. These properties suggest that it may be a potential candidate for use in agriculture [138].

Currently, there is a growing interest in the use of packaging materials based on microbial polysaccharides for storing foodstuffs and protecting perishable items from the pathogens mentioned above. In Table 5, the role of microbial polysaccharides in the composition of the packaging material is examined.

Microbial polysaccharides have the potential to be utilized in antimicrobial packaging. It is, however, important to note that the antimicrobial properties of pure microbial polysaccharides do not undergo significant improvement [74]. Consequently, additional antimicrobial agents that interact effectively with microbial polysaccharides are incorporated into such packaging materials. For instance, the research [140] indicates that carvacrol enhances the antimicrobial efficacy of packaging materials. Furthermore, polysaccharides, such as xanthan gum and curdlan gum, have not been extensively investigated as a material for antimicrobial packaging materials.

In addition to the fact that films and packages based on microbial polysaccharides are biodegradable, such packages also improve the intestinal microflora. Consequently, these materials are edible and beneficial to the intestinal microflora. In this context, it is paramount to ascertain which microbial polysaccharides should be employed to obtain the desired packaging material. Furthermore, it is essential to select the most appropriate additional polysaccharides that are compatible with microbial polysaccharides. This will enhance the structural integrity of packaging materials and reduce their cost. It is also essential to consider the role of plasticizers, which directly impact the packaging material’s properties, including its appearance, elasticity, and strength. 

## 7. Anti-Infection Properties of Microbial Polysaccharides

Microbial polysaccharides have shown significant promise in anti-infection applications due to their unique properties, such as biocompatibility, biodegradability, and the ability to interact with biological systems. For example, xanthan gum is used in wound dressings due to its excellent moisture-retentive properties and ability to form a viscous solution. These materials can create a moist environment conducive to wound healing and inhibit microbial growth, reducing the risk of infection [141]. Additionally, another microbial polysaccharide, pullulan, is effectively used for tissue engineering [142].

Some microbial polysaccharides have been shown to inhibit biofilm formation on the surface of microbial cells, a major cause of chronic infections. For instance, pullulan and its composites have antimicrobial properties and can disrupt biofilms, making them useful in preventing infections associated with medical devices and implants [143].

Furthermore, it is established that pullulan can modulate the immune system, enhancing the body’s natural defense mechanisms against infections [142]. Xanthan gum can stimulate macrophages and other immune cells, boosting the host’s ability to fight pathogens [144].

The anti-infective properties of microbial polysaccharides have significant potential for incorporation into pharmaceuticals for drug development. In this regard, study [145] examines the anti-infective properties of glycans in the development of a vaccine. It describes the effect of glycans on the protection of the main protein against human immunodeficiency virus type 1 (HIV-1). Glycans serve as a structural shield for the shell. Furthermore, the impact of microbial polysaccharide on *Clostridium difficile* cells, a causative agent of nosocomial infections that result in considerable morbidity and mortality on a global scale, is elucidated [146].

## 8. Drug Delivery Applications of Microbial Polysaccharides

Chemically modified polysaccharides are widely used in the pharmaceutical field as drug delivery systems. They are commonly used to control the release of drugs and other biologically active substances. Their structure contains many functional groups, allowing bioconjugation with other biological macromolecules such as proteins [95]. For example, they can aid in the healing of wounds in patients with diabetes mellitus [147]. Microbial polysaccharides can also be used as a material for load cells, serving as an alternative to hydrogel load cells due to several advantages, including biological purity and environmental friendliness [148].

The potential of microbial polysaccharides to revolutionize drug delivery has been demonstrated in recent studies [149]. Specifically, these polysaccharides can slow down the release of active pharmaceutical substances, which could have significant implications for the pharmaceutical industry. Pharmacokinetic simulations have shown that pullulan–dexamethasone conjugates can release free and active dexamethasone in the vitreous humor for over 16 days. Even though a significant proportion of dexamethasone is eliminated from the eye as bound pullulan–dexamethasone, it still has a sustained release effect. This makes pullulan-based drug conjugates a promising option for developing intravitreal drug delivery systems. These systems could reduce the frequency of injections and improve the delivery of drugs into retinal cells [150]. The study’s findings on the impact of microbial polysaccharides on the release process are summarized in Table 6.

Microbial polysaccharides have the key benefit of being usable as bio-inks for 3D printers, offering excellent properties [154]. Furthermore, the work of [155] demonstrated that all the developed frameworks support the attachment and proliferation of human liver cancer cells (HepG2), making them more compelling options as building materials.

There is ongoing research to explore the healing properties of thermal mud, which has been discovered to contain microbial polysaccharides. For instance, [116] studied the use of mud in Italian resorts for treating arthro-rheumatic diseases. The anti-inflammatory and antioxidant effects of microbial polysaccharides have been shown to have an impact on the affected area.

Microbial polysaccharides are increasingly used in drug delivery. Curdlan, for example, is advantageous in immunotherapy for the encapsulation of drugs within nanoparticles. When incorporated into the nanoparticle’s core, curdlan retains its immunogenicity, preventing its immunotherapeutic properties from manifesting on the nanoparticle’s surface [67]. Furthermore, alginate hydrogels have the potential to serve as a synthetic stem cell niche by providing a three-dimensional platform for creating a cell niche through the encapsulation of cells in a polymer network [134].

The work [156] illustrates that cross-linked dextran was employed to polydopamine hydrogel and demonstrated highly efficacious healing properties in both in vitro and in vivo assays. One of the principal advantages of microbial polysaccharides is that they may be employed as complementary or alternative drugs against cancer [63]. The presence of sugar monomers, such as glucose, arabinose, and galactose, within microbial polysaccharides results in these compounds exhibiting enhanced anticancer and/or antimicrobial activity [157].

Polysaccharides derived from microbes have been shown to have anti-proliferative effects on various types of tumor cells, including those in the intestines, liver, and breast. They affect tumor development through several mechanisms, such as promoting apoptosis, inducing cell cycle arrest, and exhibiting antimutagenic, antioxidant, antiangiogenic, and anti-inflammatory effects [158]. In addition to these properties, microbial polysaccharides are also highly soluble. For example, [159] demonstrated that nanogels containing polysaccharides can improve the solubility of insoluble substances like resveratrol.

Transdermal drug delivery systems in the form of patches or films not only prevent metabolism at the first pass and offer painless administration but also help patients with dysphagia, increase patient convenience, and can be used independently [160]. Curdlan and bacterial cellulose may be the most suitable in such cases due to their properties. Microbial polysaccharides can also be used to make semi-solid drug delivery forms such as ointments. These creams may have an oily texture, so pullulan and xanthan gum may be a better option.

The aforementioned microbial polysaccharides have considerable potential in the field of medicine. They can be employed as a means of delivering essential elements to specific body organs, and they also possess antimicrobial and antioxidant properties, which are their key advantages.

## 9. Commercial Applications

The production and utilization of microbial polysaccharides, including xanthan gum, curdlan gum, dextran, and others, have been extensively developed. Currently, the products resulting from microbial polysaccharide research are commonly used in many laboratories. Consequently, numerous commercial food films and coatings, such as Tal-ProLong™, FreshSeal^®^, NatureSeal^®^, ProFruit, Provegetable, Vege-Coat, Z*Coat™, Articoat DLP2, Myvacet, and MycoStop gel, are used in the food industry to extend the shelf life of food products. Although none of them included antimicrobials in the film and/or coating, they were able to extend the microbiological shelf life of various food products, possibly due to the modified atmosphere created [161]. In the cosmetics field, several brands, including Mary Kay™, Bio Enzymes™, and eLeaders™, are producing cellulose facial masks [22].

The Pfizer company (Mumbai, India) first introduced pullulan films on the commercial market in 1982. Hayashibara has granted Pfizer the right to commercialize and sell pullulan film-based oral care products in Canada and the United States under the Listerine™ brand [111].

Furthermore, there is a significant potential for using bacterial cellulose as a packaging material (coating) for mozzarella cheese. Several studies have demonstrated that cheese coated with the aforementioned material is resistant to various bacteria for a period of eight days [162].

The preliminary studies have yielded promising results concerning the potential of microbial polysaccharides for use in biomedical engineering and medicine. This suggests that microbial polysaccharides may have a promising future in this field, especially as an alternative to modern biotechnologies [160]. The successful 3D printing of biofilms containing bacterial cellulose in the form of patches has been demonstrated. In vitro release tests have shown cellulose plays an essential role in release kinetics [154].

In light of these findings, it seems reasonable to posit that microbial polysaccharides may offer a promising avenue for future applications in the field of medicine and pharmaceuticals, particularly in the context of commercial development. Furthermore, in consideration of the favorable characteristics outlined in this review, it can be postulated that microbial polysaccharides will be employed with greater frequency in the food industry.

One of the factors contributing to the utilization of microbial polysaccharides is their relatively low production cost. For instance, bacterial cellulose is generated from agro-industrial and food waste materials sourced from inexpensive resources, such as wheat straw, coconut water, litchi, cashew apple, etc. [163]. However, the cost of cellulose is lower than that of bacterial cellulose. For example, according to Sigma-Aldrich (St. Louis, MO, USA), the price of 250 mg of colloidal cellulose is 69 $US, whereas the price of 250 mg of bacterial cellulose ranges from 89 to 400 $US.

## 10. Conclusions and Perspective

The potential for the practical application of microbial polysaccharides is largely contingent upon their resistance to bacteria, their capacity for biodegradation, their favorable impact on intestinal microflora as prebiotics, and their antioxidant properties. They have been observed to exert bifunctional effects, including the capacity to absorb a diverse range of free radicals and bind free cholesterol. Furthermore, these materials can modulate the intestinal microflora, induce immunomodulation, and exert antitumor, antimicrobial, antimicrobial, and antitoxic effects, which may contribute to their promotion as potential therapeutic agents. These materials have significant potential for utilization in drug delivery systems, for example. 

Despite the evident advantages and benefits of utilizing microbial polysaccharides, they have yet to fully displace traditional non-degradable polymers from our lives. One of the most significant factors impeding the widespread use of microbial polysaccharides is the paucity of comprehensive studies investigating their antimicrobial properties, particularly the specific mechanisms underlying their effect on pathogenic bacteria. Another equally important reason for their limited use is the lack of data on the decomposition time of packaging materials under various conditions. In light of these considerations, it is evident that further research in this area should be directed towards elucidating these issues. The use of microbial polysaccharides as a packaging material is complicated by their high solubility. It seems plausible to suggest that the synthesis of modified microbial polysaccharides may prove to be a significantly more relevant avenue of research for the development of packaging materials.

The utilization of microbial polysaccharides represents a promising approach to the long-term reduction of environmental pollution and the enhancement of the functionality of materials in a range of industrial sectors. It is imperative that continuous research and development be conducted in order to gain a comprehensive understanding of these materials and to ensure their effective integration into existing and new packaging and biomedical technologies. It seems reasonable to posit that microbial polysaccharides may prove to be a significant advance in the treatment of infectious diseases. Furthermore, the production of cellulose via a cell-free system represents a novel and substantial advancement in the domain of biotechnology. These prospects have the potential to represent a significant breakthrough in the field of medical and pharmaceutical science.

## Figures and Tables

**Table 1:** Sources of several microbial polysaccharides.

Microbial Polysaccharides	Important Properties	Sources	References
Acetan	May change intermolecular interactions	*Acetobacter xylinum*	[19]
Alginate	Moisture retention	*Pseudomonas aerugina*	[20]
Alternan	High solubility and low viscosity	*Leuconostoc mesentorides*	[21]
Bacterial cellulose	High adsorption capacity and mechanical strength	*Acetobacter xylinum* *Agrobacterium* sp. *Pseudomonas* sp. *Rhizobium* sp.	[22]
Curdlan	Stabilizer	*Agrobacterium*	[23]
Dextran	Reduces blood viscosity	*Leucomostoc* sp.	[24]
Emulsan	Stabilizer	*Acinetobacter calcoaceticus*	[25]
Gellan	Gelling agent	*Alcaligenes faecalis*	[26]
Hyaluronan	Not modified after synthesis in the body	*Streptococcus* sp.	[27]
Kefiran	Emulsifier	*Lactobacillus kefiranofaciens*	[28]
Levan	Antioxidant and prebiotic	*Erwinia* sp., *Bacillus* sp.	[29]
Mutan	High thermostability	*Strep. mutans*	[30]
Succinoglucan	High viscosity	*Alcaligenes faecalis var. myxogenes*	[31]
Welan	Stable in a wide pH range	*Alcaligenes species*	[32]
Xanthan	Thickener, gelling agent, and stabilizer	*Xanthomonas campestris*	[33]

**Table 2:** A comparison of the water vapor permeability of different polysaccharides at different concentrations.

Film Composition	WVP, (g/m·s·Pa·10^−11^)	Reference
Agar-0.2%/xanthan gum-0.05%	19.0	[90]
Pectin-0.6%/alginate-0.5%/xanthan gum-0.4%	18.1	[91]
Carrageenan-0.6%/xanthan gum-0.1%/gellan gum-0.2%	31.0	[92]
Chitosan-1%/bacterial cellulose-0.04%	From 23.9 to 26.8	[83]
Bacterial cellulose-10%	From 13.5 to 31.3	[68]
Pure curdlan-5%	17.5	[61]
Chitosan/curdlan/CMC-0.25 mol/L	28.4	[93]
Pure pullulan-5%	3.64	[50]
Pullulan/carrageenan-5%	10.0	[94]

**Table 3:** The inhibition zone (mm) growth of gram-positive and gram-negative bacteria in the presence of microbial polysaccharides contain mixtures.

Mixtures	C, mg/mL	*E. coli*	*St. aureus*	*Fusarium*	*Staphylococcus*	*L. mono*	*K. pneumonia*	References
Xanthan gum–oligosaccharide	10.0	-	33.51	-	-	-	-	[117]
Xanthan gum–poly(N-vinyl imidazole) modification	-	17.50	21.30	-	-	-	-	[118]
Curdlan gum nanofibers	-	12.00	11.00	-	-	-	-	[119]
Quaternized curdlan gum	-	8.10	-	13.00	15.00	-	-	[120]
Pullulan gum and apple fiber	-	6.00	6.00	-	-	-	-	[121]
Pullulan AgNP	2.00	12.00		-	-	10.00	12.00	[7]
HMDA–Oxidized Pullulan/Sodium alginate	10.0	12.90	13.00	-	-	-	-	[55]
Gellan gum–Xanthan gum–ZnP	-	15.00	13.00	-	-	-	-	[122]
Gellan gum–Chitosan	1.00	18.00	0.00	-	-	-	17.00	[123]

**Table 4:** Antioxidant activities of microbial polysaccharides.

Biodegradable Packages	C, mg/mL	Antioxidant Activity, %	References
Pure pullulan	1.0	0.0	[121]
Pure xanthan	1.0	0.0	[127]
Pure curdlan	1.0	8.0	[58]
Pullulan and apple fiber	-	4.9	[121]
Ferulic acid-grafted curdlan	40.0	30.0	[128]
Low-molecular-weight xanthan gum	1.0	17.0	[129]
Polyvinyl alcohol–-xanthan gum	-	40.0	[115]

**Table 5:** Antimicrobial food packages with microbial polysaccharides.

Antimicrobial Packaging Type	Polysaccharide	Results	References
Packaging film	Pullulan	Cherry stored 9 days	[139]
Packaging material	Pullulan	Fish oil stored 8 days	[140]
Packaging membrane	Bacterial cellulose	Strawberry stored 5 days	[76]
Edible coating	Gellan gum	Strawberry stored 7 days	[26]

**Table 6:** Microbial polysaccharides as drug delivery system.

Microbial Polysaccharide	Drug	Release Study	Release Properties	References
Xanthan gum	Piroxicam	33% after 10 min	Phosphate buffer solution pH 7.4 (in vitro study)	[95]
Xanthan gum	Ketoconazole	63% after 10 min	Phosphate buffer solution pH 7.4 (in vitro study)	[95]
Gellan gum	Ofloxacin	50% after 4 h	In vivo study	[151]
Pullulan	Dexamethasone	100% after 16 days	In vivo study	[150]
Curdlan	Camptothecin	80% after 96 h	Phosphate buffer solution pH 7.4 (in vitro study)	[152]
Bacterial cellulose	Doxycycline hyclate	90% after 3–5 h	Phosphate buffer solution pH 6.0 (in vitro study)	[153]

## References

[1] Cazón P., Vázquez M. (2021). Improving bacterial cellulose films by ex-situ and in-situ modifications: A review. Food Hydrocoll..

[2] Rodríguez-Félix F., Corte-Tarazón J.A., Rochín-Wong S., Fernández-Quiroz J.D., Garzón-García A.M., Santos-Sauceda I., Plascencia-Martínez D.F., Chan-Chan L.H., Vásquez-López C., Barreras-Urbina C.G. (2022). Physicochemical, structural, mechanical and antioxidant properties of zein films incorporated with no-ultrafiltered and ultrafiltered betalains extract from the beetroot (*Beta vulgaris*) bagasse with potential application as active food packaging. J. Food Eng..

[3] Cazón P., Vázquez M. (2021). Bacterial cellulose as a biodegradable food packaging material: A review. Food Hydrocoll..

[4] Chiaoprakobkij N., Suwanmajo T., Sanchavanakit N., Phisalaphong M. (2020). Curcumin-Loaded Bacterial Cellulose/Alginate/Gelatin as A Multifunctional Biopolymer Composite Film. Molecules.

[5] Sun X., Zhang J. (2021). Bacterial exopolysaccharides: Chemical structures, gene clusters and genetic engineering. Int. J. Biol. Macromol..

[6] Gomes D., Costa D., Queiroz J.A., Passarinha L.A., Sousa A. (2021). A new insight in gellan microspheres application to capture a plasmid DNA vaccine from an Escherichia coli lysate. Sep. Purif. Technol..

[7] Kanmani P., Lim S.T. (2013). Synthesis and characterization of pullulan-mediated silver nanoparticles and its antimicrobial activities. Carbohydr. Polym..

[8] Matsumoto Y., Enomoto Y., Kimura S., Iwata T. (2021). Highly deformable and recoverable cross-linked hydrogels of 1,3-α-D and 1,3-β-D-glucans. Carbohydr. Polym..

[9] Leal A.R., Oliveira L.d.S., Farias L.M., Alves C.A.N., da Costa J.N., Mata P., de Sousa P.H.M. (2021). Elaboration of mixed structured fruit formulations with agar and gellan gum: Texture, physicochemical, and sensory properties. Int. J. Gastron. Food Sci..

[10] Liang Y., Qu Z., Liu M., Wang J., Zhu M., Liu Z., Li J., Zhan X., Jia F. (2020). Effect of curdlan on the quality of frozen-cooked noodles during frozen storage. J. Cereal Sci..

[11] Tiwari S., Kavitake D., Devi P.B., Shetty P.H. (2021). Bacterial exopolysaccharides for improvement of technological, functional and rheological properties of yoghurt. Int. J. Biol. Macromol..

[12] Kouhi M., Prabhakaran M.P., Ramakrishna S. (2020). Edible polymers: An insight into its application in food, biomedicine and cosmetics. Trends Food Sci. Technol..

[13] Nadzir M.M., Nurhayati R.W., Idris F.N., Nguyen M.H. (2021). Biomedical applications of bacterial exopolysaccharides: A review. Polymers.

[14] McCarthy R.R., Ullah M.W., Booth P., Pei E., Yang G. (2019). The use of bacterial polysaccharides in bioprinting. Biotechnol. Adv..

[15] Jang E.J., Padhan B., Patel M., Pandey J.K., Xu B., Patel R. (2023). Antibacterial and biodegradable food packaging film from bacterial cellulose. Food Control.

[16] Prilepskii A., Nikolaev V., Klaving A. (2023). Conductive bacterial cellulose: From drug delivery to flexible electronics. Carbohydr. Polym..

[17] Yang Y.T., Qin M.K., Sun Q., Gao Y.F., Ma C.Y., Wen J.L. (2022). Structural elucidation and targeted valorization of poplar lignin from the synergistic hydrothermal-deep eutectic solvent pretreatment. Int. J. Biol. Macromol..

[18] Abdalla A.K., Ayyash M.M., Olaimat A.N., Osaili T.M., Al-Nabulsi A.A., Shah N.P., Holley R. (2021). Exopolysaccharides as Antimicrobial Agents: Mechanism and Spectrum of Activity. Front. Microbiol..

[19] Trček J., Dogsa I., Accetto T., Stopar D. (2021). Acetan and Acetan-Like Polysaccharides: Genetics, Biosynthesis, Structure, and Viscoelasticity. Polymers.

[20] Vasudevan U.M., Lee O.K., Lee E.Y. (2021). Alginate derived functional oligosaccharides: Recent developments, barriers, and future outlooks. Carbohydr. Polym..

[21] Asgher M., Qamar S.A., Bilal M., Iqbal H.M.N. (2020). Bio-based active food packaging materials: Sustainable alternative to conventional petrochemical-based packaging materials. Food Res. Int..

[22] Fernandes I.d.A.A., Pedro A.C., Ribeiro V.R., Bortolini D.G., Ozaki M.S.C., Maciel G.M., Haminiuk C.W.I. (2020). Bacterial cellulose: From production optimization to new applications. Int. J. Biol. Macromol..

[23] Yuan M., Fu G., Sun Y., Zhang D. (2021). Biosynthesis and applications of curdlan. Carbohydr. Polym..

[24] Pacelli S., Di Muzio L., Paolicelli P., Fortunati V., Petralito S., Trilli J., Casadei M.A. (2021). Dextran-polyethylene glycol cryogels as spongy scaffolds for drug delivery. Int. J. Biol. Macromol..

[25] Yi G., Son J., Yoo J., Park C., Koo H. (2019). Emulsan-based nanoparticles for in vivo drug delivery to tumors. Biochem. Biophys. Res. Commun..

[26] Tomadoni B., Moreira M.R., Pereda M., Ponce A.G. (2018). Gellan-based coatings incorporated with natural antimicrobials in fresh-cut strawberries: Microbiological and sensory evaluation through refrigerated storage. LWT.

[27] Mai V.Q., Vo T.T., Meere M. (2018). Modelling hyaluronan degradation by streptococcus pneumoniae hyaluronate lyase. Math. Biosci..

[28] Júnior L.M., Vieira R.P., Anjos C.A.R. (2020). Kefiran-based films: Fundamental concepts, formulation strategies and properties. Carbohydr. Polym..

[29] de Siqueira E.C., Rebouças J.d.S., Pinheiro I.O., Formiga F.R. (2020). Levan-based nanostructured systems: An overview. Int. J. Pharm..

[30] Krzyściak W., Jurczak A., Kościelniak D., Bystrowska B., Skalniak A. (2013). The virulence of Streptococcus mutans and the ability to form biofilms. Eur. J. Clin. Microbiol. Infect. Dis..

[31] Omar-Aziz M., Yarmand M.S., Khodaiyan F., Mousavi M., Gharaghani M., Kennedy J.F., Hosseini S.S. (2020). Chemical modification of pullulan exopolysaccharide by octenyl succinic anhydride: Optimization, physicochemical, structural and functional properties. Int. J. Biol. Macromol..

[32] Liao K., An J., Fu L., Zhang H., Wei M., Bai J., He Y. (2022). Adsorption of Welan Gum on Montmorillonite and Its Influencing Factors. Polymers.

[33] Âa-Ochoa F.G., Santos V.E., Casas J.A., Âmez E.G. (2000). Xanthan gum: Production, recovery, and properties. Biotechnol. Adv..

[34] Lee H., Rukmanikrishnan B., Lee J. (2019). Rheological, morphological, mechanical, and water-barrier properties of agar/gellan gum/montmorillonite clay composite films. Int. J. Biol. Macromol..

[35] Sun Y., Liu Y., Li Y., Lv M., Li P., Xu H., Wang L. (2011). Preparation and characterization of novel curdlan/chitosan blending membranes for antibacterial applications. Carbohydr. Polym..

[36] Gomes G.V.P., Assis D.d.J., Da Silva J.B.A., Santos-Ebinuma V.d.C., Costa L.A.S., Druzianl J.I. (2015). Obtaining Xanthan Gum Impregnated with Cellulose Microfibrils Derived from Sugarcane Bagasse. Mater. Today Proc..

[37] Palaniraj A., Jayaraman V. (2011). Production, recovery and applications of xanthan gum by Xanthomonas campestris. J. Food Eng..

[38] Tang J.-L., Tang D.-J., Dubrow Z.E., Bogdanove A., An S.-Q. (2021). Xanthomonas campestris Pathovars. Trends Microbiol..

[39] Mohsin A., Zaman W.Q., Guo M., Ahmed W., Khan I.M., Niazi S., Rehman A., Hang H., Zhuang Y. (2020). Xanthan-Curdlan nexus for synthesizing edible food packaging films. Int. J. Biol. Macromol..

[40] Riaz T., Iqbal M.W., Jiang B., Chen J. (2021). A review of the enzymatic, physical, and chemical modification techniques of xanthan gum. Int. J. Biol. Macromol..

[41] Rafigh S.M., Soleymani A.R., Heydarinasab A. (2021). Sulfated xanthan: Synthesis, characterization and biological evaluation. Polym. Bull..

[42] Ahmed J. (2022). Optimization of high-pressure-assisted xanthan gum dispersions for the maximization of rheological moduli: Application of time-pressure/temperature superposition principle. Food Hydrocoll..

[43] Poret F., Cordinier A., Hucher N., Grisel M., Savary G. (2021). Impact of the synergistic interaction between xanthan and galactomannan on the stickiness properties of residual film after application on a surface. Carbohydr. Polym..

[44] Byram P.K., Sunka K.C., Barik A., Kaushal M., Dhara S., Chakravorty N. (2020). Biomimetic silk fibroin and xanthan gum blended hydrogels for connective tissue regeneration. Int. J. Biol. Macromol..

[45] Cazarolli J.C., Silva T.L., Ribas R.K.d.C., Costa L.d.F.X., de Moura T., Galeazzi C.F., da Rosa P.D., Kenne D.C., Carvalho R., Valente P. (2020). Deterioration potential of *Aureobasidium pullulans* on biodiesel, diesel, and B20 blend. Int. Biodeterior. Biodegrad..

[46] Kraśniewska K., Pobiega K., Gniewosz M. (2019). Pullulan-Biopolymer with Potential for Use as Food Packaging. Int. J. Food Eng..

[47] Roy S., Rhim J.W. (2021). Gelatin/agar-based functional film integrated with Pickering emulsion of clove essential oil stabilized with nanocellulose for active packaging applications. Colloids Surf. A Physicochem. Eng. Asp..

[48] Li S., Yi J., Yu X., Wang Z., Wang L. (2020). Preparation and characterization of pullulan derivative/chitosan composite film for potential antimicrobial applications. Int. J. Biol. Macromol..

[49] Singh R.S., Kaur N., Hassan M., Kennedy J.F. (2021). Pullulan in biomedical research and development—A review. Int. J. Biol. Macromol..

[50] Najafi Z., Kahn C.J.F., Bildik F., Arab-Tehrany E., Şahin-Yeşilçubuk N. (2021). Pullulan films loading saffron extract encapsulated in nanoliposomes; preparation and characterization. Int. J. Biol. Macromol..

[51] Wei X., Liu G.L., Jia S.L., Chi Z., Hu Z., Chi Z.M. (2021). Pullulan biosynthesis and its regulation in *Aureobasidium* spp.. Carbohydr. Polym..

[52] Xia S., Yu H., Qiu Y., Zhao Y., Li H., Zhang J., Zhu J. (2024). A novel curdlan/methyl cellulose/walnut green husk polyphenol edible composite film for walnut packaging. Int. J. Biol. Macromol..

[53] Alizadeh-Sani M., Ehsani A., Kia E.M., Khezerlou A. (2019). Microbial gums: Introducing a novel functional component of edible coatings and packaging. Appl. Microbiol. Biotechnol..

[54] Khaliq T., Sohail M., Shah S.A., Mahmood A., Kousar M., Jabeen N. (2022). Bioactive and multifunctional keratin-pullulan based hydrogel membranes facilitate re-epithelization in diabetic model. Int. J. Biol. Macromol..

[55] Li S., Yi J., Yu X., Wang Z., Wang L. (2020). Preparation and characterization of pullulan derivative antibacterial composite films. Mater. Sci. Eng. C.

[56] Zhai W., Iwata T. (2019). Synthesis and properties of curdlan branched and linear mixed ester derivatives. Polym. Degrad. Stab..

[57] Chen Y., Wang F. (2020). Review on the preparation, biological activities and applications of curdlan and its derivatives. Eur. Polym. J..

[58] Marubayashi H., Yukinaka K., Enomoto-Rogers Y., Takemura A., Iwata T. (2014). Curdlan ester derivatives: Synthesis, structure, and properties. Carbohydr. Polym..

[59] Verma D.K., Niamah A.K., Patel A.R., Thakur M., Sandhu K.S., Chávez-González M.L., Shah N., Aguilar C.N. (2020). Chemistry and microbial sources of curdlan with potential application and safety regulations as prebiotic in food and health. Food Res. Int..

[60] Matsumoto Y., Enomoto Y., Kimura S., Iwata T. (2021). Highly stretchable curdlan hydrogels and mechanically strong stretched-dried-gel-films obtained by strain-induced crystallization. Carbohydr. Polym..

[61] Zhang Y., Zhou L., Zhang C., Show P.L., Du A., Fu J., Ashokkumar V. (2020). Preparation and characterization of curdlan/polyvinyl alcohol/thyme essential oil blending film and its application to chilled meat preservation. Carbohydr. Polym..

[62] Su Z., Erdene-Ochir T., Ganbold T., Baigude H. (2020). Design of curdlan-based pH-sensitive polymers with endosome buffering functionality for siRNA delivery. Int. J. Biol. Macromol..

[63] Prateeksha, Sharma V.K., Liu X., Oyarzún D.A., Abdel-Azeem A.M., Atanasov A.G., Hesham A.E.-L., Barik S.K., Gupta V.K., Singh B.N. (2021). Microbial polysaccharides: An emerging family of natural biomaterials for cancer therapy and diagnostics. Semin. Cancer Biol..

[64] Basha R.Y., Venkatachalam G., Kumar T.S.S., Doble M. (2020). Dimethylaminoethyl modified curdlan nanoparticles for targeted siRNA delivery to macrophages. Mater. Sci. Eng. C.

[65] Bao M., Ehexige E., Xu J., Ganbold T., Han S., Baigude H. (2021). Oxidized curdlan activates dendritic cells and enhances antitumor immunity. Carbohydr. Polym..

[66] Qian Y., Bian L., Wang K., Chia W.Y., Khoo K.S., Zhang C., Chew K.W. (2021). Preparation and characterization of curdlan/nanocellulose blended film and its application to chilled meat preservation. Chemosphere.

[67] Ganie S.A., Rather L.J., Li Q. (2022). Review on Anti-cancer and Anti-microbial Applications of Curdlan Biomaterials. J. Polym. Environ..

[68] Cazón P., Velázquez G., Vázquez M. (2020). Bacterial cellulose films: Evaluation of the water interaction. Food Packag. Shelf Life.

[69] Zhong C. (2020). Industrial-Scale Production and Applications of Bacterial Cellulose. Front. Bioeng. Biotechnol..

[70] Khattak W.A., Ul-Islam M., Ullah M.W., Yu B., Khan S., Park J.K. (2014). Yeast cell-free enzyme system for bio-ethanol production at elevated temperatures. Process Biochem..

[71] Kim Y., Ullah M.W., Ul-Islam M., Khan S., Jang J.H., Park J.K. (2019). Self-assembly of bio-cellulose nanofibrils through intermediate phase in a cell-free enzyme system. Biochem. Eng. J..

[72] Ullah M.W., Ul-Islam M., Khan S., Kim Y., Park J.K. (2015). Innovative production of bio-cellulose using a cell-free system derived from a single cell line. Carbohydr. Polym..

[73] Ullah M.W., Ul-Islam M., Khan S., Kim Y., Park J.K. (2016). Structural and physico-mechanical characterization of bio-cellulose produced by a cell-free system. Carbohydr. Polym..

[74] Lahiri D., Nag M., Dutta B., Dey A., Sarkar T., Pati S., Edinur H.A., Kari Z.A., Noor N.H.M., Ray R.R. (2021). Bacterial cellulose: Production, characterization and application as antimicrobial agent. Int. J. Mol. Sci..

[75] Wang J., Tavakoli J., Tang Y. (2019). Bacterial cellulose production, properties and applications with different culture methods—A review. Carbohydr. Polym..

[76] El-Gendi H., Salama A., El-Fakharany E.M., Saleh A.K. (2023). Optimization of bacterial cellulose production from prickly pear peels and its ex situ impregnation with fruit byproducts for antimicrobial and strawberry packaging applications. Carbohydr. Polym..

[77] Liu F., Chen C., Qian J. (2021). Film-like bacterial cellulose/cyclodextrin oligomer composites with controllable structure for the removal of various persistent organic pollutants from water. J. Hazard. Mater..

[78] Cheng F., Xu L., Dai J., Yi X., He J., Li H. (2022). N, O-carboxymethyl chitosan/oxidized cellulose composite sponge containing ε-poly-L-lysine as a potential wound dressing for the prevention and treatment of postoperative adhesion. Int. J. Biol. Macromol..

[79] Ojagh S.M.A., Vahabzadeh F., Karimi A. (2021). Synthesis and characterization of bacterial cellulose-based composites for drug delivery. Carbohydr. Polym..

[80] Mbituyimana B., Liu L., Ye W., Boni B.O.O., Zhang K., Chen J., Thomas S., Vasilievich R.V., Shi Z., Yang G. (2021). Bacterial cellulose-based composites for biomedical and cosmetic applications: Research progress and existing products. Carbohydr. Polym..

[81] Ma T., Lv L., Ouyang C., Hu X., Liao X., Song Y., Hu X. (2021). Rheological behavior and particle alignment of cellulose nanocrystal and its composite hydrogels during 3D printing. Carbohydr. Polym..

[82] Ghozali M., Meliana Y., Chalid M. (2021). Synthesis and characterization of bacterial cellulose by Acetobacter xylinum using liquid tapioca waste. Mater. Today Proc..

[83] Salari M., Khiabani M.S., Mokarram R.R., Ghanbarzadeh B., Kafil H.S. (2021). Use of gamma irradiation technology for modification of bacterial cellulose nanocrystals/chitosan nanocomposite film. Carbohydr. Polym..

[84] Ju S., Zhang F., Duan J., Jiang J. (2020). Characterization of bacterial cellulose composite films incorporated with bulk chitosan and chitosan nanoparticles: A comparative study. Carbohydr. Polym..

[85] Zhang Y., Chen G., Qin W., Men X., Liu L., Zhang Y., Li Q., Wang L., Zhang H. (2023). In Situ Fermentation of an Ultra-Strong, Microplastic-Free, and Biodegradable Multilayer Bacterial Cellulose Film for Food Packaging. ACS Appl. Mater. Interfaces.

[86] Wei Z., Pan P., Hong F.F., Cao Z., Ji Y., Chen L. (2021). A novel approach for efficient fabrication of chitosan nanoparticles-embedded bacterial nanocellulose conduits. Carbohydr. Polym..

[87] Morais E.S., Silva N.H., Sintra T.E., Santos S.A., Neves B.M., Almeida I.F., Costa P.C., Correia-Sá I., Ventura S.P., Silvestre A.J. (2019). Anti-inflammatory and antioxidant nanostructured cellulose membranes loaded with phenolic-based ionic liquids for cutaneous application. Carbohydr. Polym..

[88] Pang Z., Bourouis I., Sun M., Cao J., Liu P., Sun R., Chen C., Li H., Liu X. (2022). Physicochemical properties and microstructural behaviors of rice starch/soy proteins mixtures at different proportions. Int. J. Biol. Macromol..

[89] Tarique J., Sapuan S.M., Khalina A. (2021). Effect of glycerol plasticizer loading on the physical, mechanical, thermal, and barrier properties of arrowroot (*Maranta arundinacea*) starch biopolymers. Sci. Rep..

[90] Rukmanikrishnan B., Rajasekharan S.K., Lee J., Lee J. (2019). Biocompatible agar/xanthan gum composite films: Thermal, mechanical, UV, and water barrier properties. Polym. Adv. Technol..

[91] Fan Y., Yang J., Duan A., Li X. (2021). Pectin/sodium alginate/xanthan gum edible composite films as the fresh-cut package. Int. J. Biol. Macromol..

[92] Balasubramanian R., Kim S.S., Lee J., Lee J. (2019). Effect of TiO_2_ on highly elastic, stretchable UV protective nanocomposite films formed by using a combination of k-Carrageenan, xanthan gum and gellan gum. Int. J. Biol. Macromol..

[93] Wang K., Du L., Zhang C., Lu Z., Lu F., Zhao H. (2019). Preparation of chitosan/curdlan/carboxymethyl cellulose blended film and its characterization. J. Food Sci. Technol..

[94] Roy S., Rhim J.-W. (2020). Fabrication of Copper Sulfide Nanoparticles and Limonene Incorporated Pullulan/Carrageenan-Based Film with Improved Mechanical and Antibacterial Properties. Polymers.

[95] Dimofte A., Dinu M.V., Anghel N., Doroftei F., Spiridon I. (2022). Xanthan and alginate-matrix used as transdermal delivery carrier for piroxicam and ketoconazole. Int. J. Biol. Macromol..

[96] Mostafavi F.S., Zaeim D. (2020). Agar-based edible films for food packaging applications—A review. Int. J. Biol. Macromol..

[97] Kuthiala T., Thakur K., Sharma D., Singh G., Khatri M., Arya S.K. (2022). The eco-friendly approach of cocktail enzyme in agricultural waste treatment: A comprehensive review. Int. J. Biol. Macromol..

[98] Ibrahim S., Elsayed H., Hasanin M. (2021). Biodegradable, Antimicrobial and Antioxidant Biofilm for Active Packaging Based on Extracted Gelatin and Lignocelluloses Biowastes. J. Polym. Environ..

[99] Sintim H.Y., Bary A.I., Hayes D.G., Wadsworth L.C., Anunciado M.B., English M.E., Bandopadhyay S., Schaeffer S.M., DeBruyn J.M., Miles C.A. (2020). In situ degradation of biodegradable plastic mulch films in compost and agricultural soils. Sci. Total Environ..

[100] Tazhibayeva S., Tyussyupova B., Yermagambetova A., Kokanbayev A., Musabekov K. (2020). Preparation and regulation of structuralmechanical properties of biodegradable films based on starch and agar. East.-Eur. J. Enterp. Technol..

[101] Spada J.C., Jasper A., Tessaro I.C. (2020). Biodegradable Cassava Starch Based Foams Using Rice Husk Waste as Macro Filler. Waste Biomass Valorization.

[102] Janczak K., Dąbrowska G.B., Raszkowska-Kaczor A., Kaczor D., Hrynkiewicz K., Richert A. (2020). Biodegradation of the plastics PLA and PET in cultivated soil with the participation of microorganisms and plants. Int. Biodeterior. Biodegrad..

[103] Guzman-Puyol S., Hierrezuelo J., Benítez J.J., Tedeschi G., Porras-Vázquez J.M., Heredia A., Athanassiou A., Romero D., Heredia-Guerrero J.A. (2022). Transparent, UV-blocking, and high barrier cellulose-based bioplastics with naringin as active food packaging materials. Int. J. Biol. Macromol..

[104] Guo J., Ge L., Li X., Mu C., Li D. (2014). Periodate oxidation of xanthan gum and its crosslinking effects on gelatin-based edible films. Food Hydrocoll..

[105] Hazirah M.A.S.P.N., Isa M.I.N., Sarbon N.M. (2016). Effect of xanthan gum on the physical and mechanical properties of gelatin-carboxymethyl cellulose film blends. Food Packag Shelf Life.

[106] Guo T., Wang W., Song J., Jin Y., Xiao H. (2021). Dual-responsive carboxymethyl cellulose/dopamine/cystamine hydrogels driven by dynamic metal-ligand and redox linkages for controllable release of agrochemical. Carbohydr. Polym..

[107] Chen F., Chi C. (2021). Development of pullulan/carboxylated cellulose nanocrystal/tea polyphenol bionanocomposite films for active food packaging. Int. J. Biol. Macromol..

[108] Kim J.Y., Choi Y.G., Kim S.R.B., Lim S.T. (2014). Humidity stability of tapioca starch-pullulan composite films. Food Hydrocoll..

[109] Freitas F., Alves V.D., Reis M.A., Crespo J.G., Coelhoso I.M. (2013). Microbial polysaccharide-based membranes: Current and future applications. J. Appl. Polym. Sci..

[110] Omar-Aziz M., Khodaiyan F., Yarmand M.S., Mousavi M., Gharaghani M., Kennedy J.F., Hosseini S.S. (2021). Combined effects of octenylsuccination and beeswax on pullulan films: Water-resistant and mechanical properties. Carbohydr. Polym..

[111] Trinetta V., Cutter C.N. (2016). Pullulan: A Suitable Biopolymer for Antimicrobial Food Packaging Applications. Antimicrob. Food Packag..

[112] Trinetta V., Cutter C.N., Floros J.D. (2011). Effects of ingredient composition on optical and mechanical properties of pullulan film for food-packaging applications. LWT—Food Sci. Technol..

[113] Mohsin A., Sun J., Khan I.M., Hang H., Tariq M., Tian X., Ahmed W., Niazi S., Zhuang Y., Chu J. (2019). Sustainable biosynthesis of curdlan from orange waste by using Alcaligenes faecalis: A systematically modeled approach. Carbohydr. Polym..

[114] Wong L.C., Leh C.P., Goh C.F. (2021). Designing cellulose hydrogels from non-woody biomass. Carbohydr. Polym..

[115] Raschip I.E., Fifere N., Varganici C.-D., Dinu M.V. (2020). Development of antioxidant and antimicrobial xanthan-based cryogels with tuned porous morphology and controlled swelling features. Int. J. Biol. Macromol..

[116] Zampieri R.M., Adessi A., Caldara F., De Philippis R., Valle L.D., La Rocca N. (2022). In vivo anti-inflammatory and antioxidant effects of microbial polysaccharides extracted from Euganean therapeutic muds. Int. J. Biol. Macromol..

[117] Wang Z., Yang Q., Wang X., Li R., Qiao H., Ma P., Sun Q., Zhang H. (2020). Antibacterial activity of xanthan-oligosaccharide against Staphylococcus aureus via targeting biofilm and cell membrane. Int. J. Biol. Macromol..

[118] Elella M.H.A., Mohamed R.R., ElHafeez E.A., Sabaa M.W. (2017). Synthesis of novel biodegradable antibacterial grafted xanthan gum. Carbohydr. Polym..

[119] Lin M., Long H., Liang M., Chu B., Ren Z., Zhou P., Wu C., Liu Z., Wang Y. (2021). Antifracture, Antibacterial, and Anti-inflammatory Hydrogels Consisting of Silver-Embedded Curdlan Nanofibrils. ACS Appl. Mater. Interfaces.

[120] Chen M., Liang P. (2017). Synthesis and antibacterial activity of quaternized curdlan. Polym. Bull..

[121] Luís Â., Ramos A., Domingues F. (2021). Pullulan–Apple Fiber Biocomposite Films: Optical, Mechanical, Barrier, Antioxidant and Antibacterial Properties. Polymers.

[122] Rukmanikrishnan B., Ismail F.R.M., Manoharan R.K., Kim S.S., Lee J. (2020). Blends of gellan gum/xanthan gum/zinc oxide based nanocomposites for packaging application: Rheological and antimicrobial properties. Int. J. Biol. Macromol..

[123] Novac O., Lisa G., Profire L., Tuchilus C., Popa M.I. (2014). Antibacterial quaternized gellan gum based particles for controlled release of ciprofloxacin with potential dermal applications. Mater. Sci. Eng. C.

[124] Kumar A., Saini C.S. (2021). Edible composite bi-layer coating based on whey protein isolate, xanthan gum and clove oil for prolonging shelf life of tomatoes. Meas. Food.

[125] Alaei S., Omidi Y., Omidian H. (2021). In vitro evaluation of adhesion and mechanical properties of oral thin films. Eur. J. Pharm. Sci..

[126] Feng X., Wang W., Chu Y., Gao C., Liu Q., Tang X. (2020). Effect of cinnamon essential oil nanoemulsion emulsified by OSA modified starch on the structure and properties of pullulan based films. LWT.

[127] Rather S.A., Masoodi F.A., Rather J.A., Akhter R., Gani A., Ganaie T.A. (2021). Effects of xanthan gum, canning and storage period on fatty acid profile and cholesterol oxidation of restructured low-fat meat product of India. Food Chem..

[128] Cai W.-D., Zhu J., Wu L.-X., Qiao Z.-R., Li L., Yan J.-K. (2019). Preparation, characterization, rheological and antioxidant properties of ferulic acid-grafted curdlan conjugates. Food Chem..

[129] Hu X., Wang K., Yu M., He P., Qiao H., Zhang H., Wang Z. (2019). Characterization and Antioxidant Activity of a Low-Molecular-Weight Xanthan Gum. Biomolecules.

[130] de Abreu G.F., Batista L.L., Adeodato D.C., de Albuquerque A.V., Ferraz-Carvalho R.S., de Lima R.P., de Souza V.S., de Carvalho G.L., LA Aguiar J. (2020). Use of bacterial cellulose film for repair of bile duct injury in pigs. J. Biomater. Appl..

[131] Gumienna M., Górna B. (2021). Antimicrobial Food Packaging with Biodegradable Polymers and Bacteriocins. Molecules.

[132] Shi Y., Liu J., Yan Q., You X., Yang S., Jiang Z. (2018). In vitro digestibility and prebiotic potential of curdlan (1 → 3)-β- d -glucan oligosaccharides in Lactobacillus species. Carbohydr. Polym..

[133] Xu J., Wang R., Zhang H., Wu J., Zhu L., Zhan X. (2021). In vitro assessment of prebiotic properties of oligosaccharides derived from four microbial polysaccharides. LWT.

[134] Lee J.W., Kim H.S., Yon S.J., Matsumoto T., Lee S.K., Lee K.Y. (2022). In vitro culture of hematopoietic stem cell niche using angiopoietin-1-coupled alginate hydrogel. Int. J. Biol. Macromol..

[135] Sandoval J.L.S., Fonseca P.E.R., Arévalo A.O.H., Sira E.E.P., Ricci J., Dufour D. (2021). Development and Characterization of Edible Films from Chachafruto (Erythrina edulis Triana) Starch. Starch/Staerke.

[136] Lin C., Chen B., Liu Y., Chen Y., Liu M., Zhu J.Y. (2021). Carboxylated cellulose nanocrystals with chiral nematic property from cotton by dicarboxylic acid hydrolysis. Carbohydr. Polym..

[137] Wilk S., Benko A. (2021). Advances in fabricating the electrospun biopolymer-based biomaterials. J. Funct. Biomater..

[138] El-Naggar M.E., Hasanin M., Youssef A.M., Aldalbahi A., El-Newehy M.H., Abdelhameed R.M. (2020). Hydroxyethyl cellulose/bacterial cellulose cryogel dopped silver@titanium oxide nanoparticles: Antimicrobial activity and controlled release of Tebuconazole fungicide. Int. J. Biol. Macromol..

[139] Zhang L., Wu J., Shen Z., Zhang H., Zhan X. (2023). Arginine-carboxylated pullulan, a potential antibacterial material for food packaging. Biomater. Adv..

[140] Ertan K., Celebioglu A., Chowdhury R., Sumnu G., Sahin S., Altier C., Uyar T. (2023). Carvacrol/cyclodextrin inclusion complex loaded gelatin/pullulan nanofibers for active food packaging applications. Food Hydrocoll..

[141] Tang S., Gong Z., Wang Z., Gao X., Zhang X. (2022). Multifunctional hydrogels for wound dressings using xanthan gum and polyacrylamide. Int. J. Biol. Macromol..

[142] Manivannan M., Nathan S.S., Sasikumar P., Ramkumar L., Navaneethan D., Prabu P., Anjalin F.M., Dharamarj N., Alqahtani M.S., Abbas M. (2023). Review on applications of Pullulan in bone tissue engineering: Blends and composites with natural and synthetic polymers. Polym. Polym. Compos..

[143] Roy S., Halder M., Ramprasad P., Dasgupta S., Singh Y., Pal D. (2023). Oxidized pullulan exhibits potent antibacterial activity against S. aureus by disrupting its membrane integrity. Int. J. Biol. Macromol..

[144] Liu F., Zhang X., Ling P., Liao J., Zhao M., Mei L., Shao H., Jiang P., Song Z., Chen Q. (2017). Immunomodulatory effects of xanthan gum in LPS-stimulated RAW 264.7 macrophages. Carbohydr. Polym..

[145] Seabright G.E., Doores K.J., Burton D.R., Crispin M. (2019). Protein and Glycan Mimicry in HIV Vaccine Design. J. Mol. Biol..

[146] Martin C.E., Broecker F., Oberli M.A., Komor J., Mattner J., Anish C., Seeberger P.H. (2013). Immunological Evaluation of a Synthetic Clostridium difficile Oligosaccharide Conjugate Vaccine Candidate and Identification of a Minimal Epitope. J. Am. Chem. Soc..

[147] Pang L., Liao Q., Zou L., Zhang C., Nie X., Yi Z., Fu C., Zhang J. (2022). Two glycoproteins from medicinal insect *Periplaneta americana* (L.) promote diabetic wound healing via macrophage polarization modulation. Int. J. Biol. Macromol..

[148] Zaidi S.F.A., Kim Y.A., Saeed A., Sarwar N., Lee N.-E., Yoon D.H., Lim B., Lee J.H. (2022). Tannic acid modified antifreezing gelatin organohydrogel for low modulus, high toughness, and sensitive flexible strain sensor. Int. J. Biol. Macromol..

[149] Zhang X., Wei D., Xu Y., Zhu Q. (2021). Hyaluronic acid in ocular drug delivery. Carbohydr. Polym..

[150] Kicková E., Sadeghi A., Puranen J., Tavakoli S., Sen M., Ranta V.-P., Arango-Gonzalez B., Bolz S., Ueffing M., Salmaso S. (2022). Pharmacokinetics of pullulan–dexamethasone conjugates in retinal drug delivery. Pharmaceutics.

[151] Das M., Giri T.K. (2020). Hydrogels based on gellan gum in cell delivery and drug delivery. J. Drug Deliv. Sci. Technol..

[152] Liu Q., Yang D., Shang T., Guo L., Yang B., Xu X. (2020). Chain conformation transition induced host-guest assembly between triple helical curdlan and: β-CD for drug delivery. Biomater. Sci..

[153] Weyell P., Beekmann U., Küpper C., Dederichs M., Thamm J., Fischer D., Kralisch D. (2019). Tailor-made material characteristics of bacterial cellulose for drug delivery applications in dentistry. Carbohydr. Polym..

[154] Olmos-Juste R., Alonso-Lerma B., Pérez-Jiménez R., Gabilondo N., Eceiza A. (2021). 3D printed alginate-cellulose nanofibers based patches for local curcumin administration. Carbohydr. Polym..

[155] Baniasadi H., Kimiaei E., Polez R.T., Ajdary R., Rojas O.J., Österberg M., Seppälä J. (2022). High-resolution 3D printing of xanthan gum/nanocellulose bio-inks. Int. J. Biol. Macromol..

[156] Zhang M., Jiang S., Han F., Li M., Wang N., Liu L. (2021). Anisotropic cellulose nanofiber/chitosan aerogel with thermal management and oil absorption properties. Carbohydr. Polym..

[157] Genc B., Taskin M., Adiguzel A. (2021). Exopolysaccharide of *Anoxybacillus pushchinoensis* G11 has antitumor and antibiofilm activities. Arch. Microbiol..

[158] Wu J., Zhang Y., Ye L., Wang C. (2021). The anti-cancer effects and mechanisms of lactic acid bacteria exopolysaccharides in vitro: A review. Carbohydr. Polym..

[159] Buosi F.S., Alaimo A., Di Santo M.C., Elías F., Liñares G.G., Acebedo S.L., Cataña M.A.C., Spagnuolo C.C., Lizarraga L., Martínez K.D. (2020). Resveratrol encapsulation in high molecular weight chitosan-based nanogels for applications in ocular treatments: Impact on human ARPE-19 culture cells. Int. J. Biol. Macromol..

[160] Laubach J., Joseph M., Brenza T., Gadhamshetty V., Sani R.K. (2021). Exopolysaccharide and biopolymer-derived films as tools for transdermal drug delivery. J. Control. Release.

[161] Raybaudi-Massilia R., Mosqueda-Melgar J., Soliva-Fortuny R., Martín-Belloso O. (2016). Combinational Edible Antimicrobial Films and Coatings. Antimicrob. Food Packag..

[162] Azhdari S., Moradi M. (2022). Application of antimicrobial coating based on carboxymethyl cellulose and natamycin in active packaging of cheese. Int. J. Biol. Macromol..

[163] Khan S., Ul-Islam M., Fatima A., Manan S., Khattak W.A., Ullah M.W., Yang G. (2023). Potential of Food and Agro-Industrial Wastes for Cost-Effective Bacterial Cellulose Production: An Updated Review of Literature. ES Food Agrofor..

